# Treatment of Acute Promyelocytic Leukemia with Single-Agent Arsenic Trioxide

**DOI:** 10.4084/MJHID.2011.056

**Published:** 2011-11-28

**Authors:** Vikram Mathews, Ezhilarasi Chendamarai, Biju George, Auro Viswabandya, Alok Srivastava

**Affiliations:** Department of Haematology, Christian Medical College and Hospital, Vellore, India

## Abstract

It is well recognized that arsenic trioxide (ATO) is an efficacious agent for the treatment of acute promyelocytic leukemia (APL). Use of single agent ATO in the treatment of APL leads to remissions which are durable in the majority. ATO is probably the most effective single agent in the treatment of APL and there have been very few reports of primary resistance. It has been used both as a single agent and in combination with other conventional drugs to treat APL. Use of ATO is the accepted standard of care in the management of relapsed APL, where it is often used effectively as a bridge to a stem cell transplant. However, its role in newly diagnosed APL remains controversial. ATO probably has multiple mechanisms of action. Better understanding of its mechanisms of action/s is likely to lead to more rationale use of this agent or its derivatives either alone or in combination with other drugs. There is limited data on the kinetics of leukemia clearance and normal haematopoietic recovery after the administration of single agent ATO for the treatment of APL, preliminary data suggests that it is likely to be different from conventional therapy. There have been a number of concerns of the potential short and long term toxicity of this agent. Most such concerns arise from the toxicity profile noted in people exposed to long term arsenic exposure in the environment. With the therapeutic doses and schedules of administration of ATO in the treatment of malignancies the overall toxicity profile has been favorable. In a resource constrained environments the use of a single agent ATO based regimen is a realistic and acceptable option to treat almost all patients. In the developed world it has the potential in combination with other agents to improve the clinical outcome with reduction of dose intensity of chemotherapy and remains an option for patients who would not tolerate conventional therapy. In this review we focus on the use of single agent ATO for the treatment of APL and summarize our experience and review the literature.

## Introduction

Arsenical compounds were used as early as 2000 BC, both as a medicine and as a poison^1^. The use of these compounds as medicines was familiar to the early physicians such as Hippocrates (460 – 377 BC), Aristotle (384 – 322 BC) and Pliny the Elder (23 – 79 AD). It was Paracelsus (1493 – 1541 AD) who used arsenicals extensively and was quoted as saying “All substances are poisons; the right dose differentiates a poison from a remedy”.^1^ In the eighteenth century, Fowlers solution (1% potassium arsenite) was very popular and was used in the treatment of various ailments, predominantly for dermatological conditions.^1^

Historically the prominence of arsenic trioxide (ATO) in the treatment of acute promyelocytic leukemia (APL) followed the observation of Chinese investigators at Harbin Medical University who systematically studied the role of arsenic based traditional Chinese recipe called ‘Ailing I,’ that had been reported to be useful in the treatment of various malignancies. They labeled this native preparation 713 (for the year and month that the study was initiated) and studied it more than a 1000 patients with various malignancies^2^. They soon noted that this agent worked best in the treatment of patients with APL. Two subsequent Chinese studies confirmed the benefit of this agent in APL.^3^,^4^

Since then there have been numerous reports on the use of ATO in the treatment of relapsed and newly diagnosed cases of APL. In this review we focus mainly on the treatment of APL with single agent ATO. We review our centers experience from 1998 and attempt to put this into context of current international management strategies, experience from other centers and address the socio-economic relevance of this strategy. We also attempt to highlight the significant differences in this approach compared to that when chemotherapeutic agents are used up front. Very briefly we also address the mechanism of action of this agent, the pharmacokinetic data and toxicity profile which we feel is critical to this discussion.

## Mechanism of Action of Arsenic Trioxide

The mechanism by which ATO induces remission in APL is still under evaluation. Table 1 summarizes the postulated mechanisms by which ATO mediates its anti-leukemia effect. While some aspects of its cellular effects are clear, the molecular basis, for these protean effects are not fully defined. Initial in-vitro studies suggested that it induces apoptosis in APL (NB4) cell lines by down regulation of Bcl-2.^5^ However, the clinical observation that a leucocytic response followed the administration of ATO consistently in the majority of patients, was suggestive of a differentiating process, as seen following the use of all-trans retinoic acid (ATRA). It was subsequently reported that in fact a dual effect of ATO was seen on promyelocytic cell lines, at low doses (0.1 – 0.5 umol/lt) there was partial differentiation and at higher doses there was preferential apoptosis (0.5 – 2 umol/lt).^6^ This has been subsequently demonstrated by a number of other groups independently.^7^,^8^ The differentiation with ATO is incomplete and usually proceeds only till the myelocyte stage following which it appears that apoptosis is the predominant mode of action.^6^ More recently data suggests that ATO but not ATRA can eliminate the leukemia initiating compartment in APL.^9^,^10^ This could partly explain why ATO but not ATRA, as single agent, is able to induce durable remission in the clinic.

## Pharmacokinetics, Pharmacogenomics, Dose and Schedule of Arsenic Trioxide

The lethal dose recorded in the literature is a single dose of more than 100mg^11^. The dose of arsenic trioxide in the initial published study by Zhi-Xiang et al^11^ was 10 mg a day for adults till complete hematological remission (CR) was achieved. Subsequently a break of 30 days was given and a second course of 28 days administered. It is important to recognize that this dosing was based on there earlier experience with doses used in native Chinese medicine and not on phase I clinical trials addressing dose limiting toxicity. The study reported by Soignet et al^12^ used a similar dose for adults but used a dose of 0.15 mg/kg/day for children. From there experience they noted that ATO is active in APL from 0.06mg/kg to 0.2 mg/kg. Within this range they did not find any difference in efficacy. Subsequent studies have used similar dosages of ATO. Pharmacokinetic studies done at this dosage demonstrated that mean peak plasma levels of 6.85 micro moles/Lt (range: 5.54 – 7.30) was achieved. The plasma half life was 12.13±3.31 hours. Importantly these parameters did not change with continuous administration.^11^ Reports of daily urinary excretion in the literature varies from between 1 – 8% to 32–65% of the daily dose administered and more importantly is continued even after the drug administration had been stopped.^11^,^13^ There is limited data on the dose and scheduling of ATO in the event of significant renal failure or for patients on dialysis.^14^ While the cumulative level of arsenic increases in the body (as demonstrated in hair and nail analysis) with continuous administration, the urinary excretion continues even after the ATO administration has stopped leading to a gradual decrease in the cumulative amount of arsenic trioxide in the body. In our own experience there was no significant difference in the ATO content from patients and normal controls hair and nail samples on long term follow up.^15^ This was the rationale for giving 4 week breaks between the courses of ATO in the regimen used by us,^16^ the breaks were intended to reduce the cumulative dose significantly.

**Figure 1:**
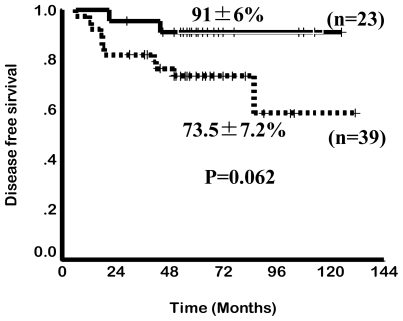
Five year Kaplan-Meier product limit estimate of disease free survival in the group that had hepatotoxicity, n=23 and those that did not, n=39.

This pattern of ATO exposure is very different from that seen with environmental toxicity where there is a slow but constant accumulation of arsenic which leads to a toxicity profile that is very different from that seen when ATO is used in therapy at currently recommended doses and schedules. Extrapolating and anticipating the toxicity profile seen with chronic environmental exposure to the potential toxicity with currently used dosage schedules of ATO is unfair, unwarranted and without any scientific basis. In the absence of a dose defining phase I clinical trial there is insufficient data on the upper limit of a safe therapeutic dose. It is of interest to note that in our initial cohort we noted a decreased risk of relapse among patient who had hepatotoxicity Vs. those that did not following treatment with ATO^17^ (Figure 1). This would suggest that there is either a significant inter-individual variation in biotransformation of this agent and as a result some patients were receiving less than an optimal dose or that there were yet unknown variables that resulted in this association.^17^ There is a need to revisit what is the optimal dose of ATO to treat APL in a large clinical trial. There is very limited data on the optimal duration of administration of ATO as a single agent in the management of ATO. Based on the general consensus that maintenance was required in the management of APL we arbitrarily opted for a 6 months duration of maintenance.^16^ Recently published data from Iran suggests that 4 courses of ATO was significantly better than 2.^18^ We subsequently undertook a larger multi-center study and randomized patients with the same induction and consolidation into a 6 and a 12 month maintenance regimen and did not find a significant benefit from increasing the duration of maintenance (unpublished data, to be presented at ASH 2011). Zhou et al reported treating children with ATO for prolonged periods of up to 3 years with very good efficacy and without significantly increasing the toxicity.^19^

It has been noted that there is considerable inter individual variation in susceptibility to ATO induced toxicity, which is probably related to differences in in-vivo biotransformation of arsenic. This in turn could be a result of age, nutritional status, co-morbid conditions, environmental factors and genetic polymorphisms.^20^ In addition to a poorly characterized arsenic methyltransferase, a number of other enzyme systems and polymorphisms have been shown to have an effect on arsenic methylation.^20^,^21^ Of these, polymorphism in the methylene tetra-hydrofolate reductase (MTHFR) gene, which results in MTHFR deficiency in 10–20% of the Caucasian population, has been reported to be associated with increased arsenic related neurotoxicity.^22^ The glutathione S-transferases (GST) are a family of proteins that conjugate glutathione (GSH) to various electrophiles.^23^ Chiou *et al* reported that genetic polymorphisms of GST M1 and GST T1 altered the methylation of arsenic.^24^ GST’s catalyze the GSH dependent reduction of hydroperoxides to their corresponding alcohols and help prevent propagation of free radicals. It is conceivable that genetic polymorphisms in these genes could alter the biotransformation of ATO, which in turn could have an impact on the efficacy and toxicity profile of this drug. We had earlier reported that the hepatotoxicity profile in a cohort of patients with newly diagnosed APL treated at our center with a single agent ATO regimen was significantly associated with the homozygous mutant of MTHFR 1298 (C/C) (RR=8.75, p=0.004) and there was a trend towards an increased risk of hepatotoxicity associated with the GST M1 null genotype (RR=3.28, p=0.06).^17^ We had hypothesized then that alteration in biotransformation possibly leads to quantitative and qualitative differences in the methylated intermediaries that are generated; these differences could have a bearing on the efficacy and toxicity profile of ATO. A recent study, in part, validates this hypothesis by suggesting that dimethylarsinous acid is more toxic than inorganic ATO and monomethylyarsinic acid,^25^ these are some of the methylated intermediaries produced in-vivo in humans and animals. It is potentially possible to consider in future the use of pure or combination of methylated ATO derivatives with optimal therapeutic and toxicity profiles.

## Clinical Experience with the Use Of Single Agent Arsenic Trioxide in Acute Promyelocytic Leukemia

The earliest clinical data available on the use of arsenic trioxide in the treatment of acute promyelocytic leukemia is from two publications from China.^26^,^27^ In these studies the complete hematological remission rate (CR) achieved varied from 65.6% to 84% and long term survival (>10 years) was seen in 9/32 patients in one of these studies.^11^ Majority of the early trials involved relapsed cases of APL. There is limited data on the use of this as a single agent in the management of newly diagnosed cases of APL. Even when used as a single agent for induction chemotherapy the subsequent consolidation therapy varies making comparisons between the published data difficult to interpret.

Our early experience with ATO consisted of two patients who were treated in the early 1990’s with what was then considered standard of care regimens, one with ATRA and one without. Both these patients relapsed and were sent on palliative care considering the therapeutic options were limited, that the options were associated with poor clinical outcome and that they were very expensive. These patients subsequently took treatment from an ayurvedic (indigenous Indian medicine) practitioner and went into durable remissions. We were aware that the agent used by the practitioner contained ATO. We were however, not sure of the dose used. The therapeutic ayurvedic mix was administered continuously in these cases and for more than 5 years in one case. One of these patients developed severe arsenic keratosis and died of a secondary squamous epithelial carcinoma.^28^

It was only after the publication in 1997 by Shen et al that we had a sense of the dose of pure ATO that could be used in humans.^11^ In 1998, we initiated a study using single agent ATO to treat APL, with intravenous ATO being manufactured in house in our hospital pharmacy (the basic cost without overheads per 10mg vial was Indian Rupees 20 or approximately US 50 cents), with appropriate quality control measures. Due to legal related issues we transferred this manufacturing process to the industry in 2001 (INTAS pharmaceuticals Ltd, Matoda, Gujarat, India). Our observation was that there was no significant difference with the agent prepared by us or that subsequently manufactured by industry in terms of infusion related toxicity or efficacy (unpublished data).

**Figure 2:**
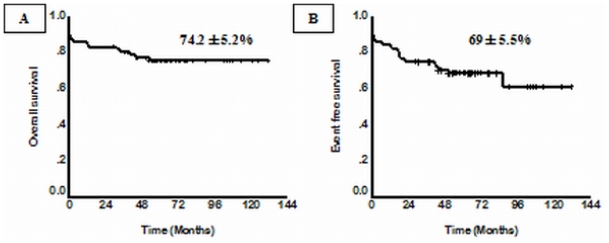
Five year Kaplan-Meier product limit estimate of (A) Overall survival of (n=72) (B) Event free survival (n=72).

From January 1998 to December 2004, 72 newly diagnosed cases of APL were treated with a regimen of single agent ATO at our center. The details of the regimen were as previously reported.^15^,^16^ Overall 62 (86.1%) achieved a hematological remission (CR). A total of 13 patients relapsed. At a median follow up 60 months, the 5 year Kaplan-Meier estimate of EFS, DFS and OS was 69±5.5%, 80±5.2% and 74.2±5.2% respectively (Figure 2). This data has since been validated by a subsequent multi-center trial in India involving 7 centers across the country (unpublished data to be presented at ASH 2011). The data from other major studies using either single agent ATO or a ATO as a major component in front line therapy^18^,^19^,^29^–^31^ is summarized in table 2.

## Toxicity Profile of Arsenic Trioxide. the Theoretical Hysteria Versus the Actual Reality!

The toxicity profile in the initial series reported by us was mild, in the majority.^16^ Significantly, there were no infusion related toxicities, alopecia or evidence of exacerbation of coagulopathy. Post induction, almost all patients for the rest of the duration of treatment had ECOG performance scores of 0 or 1. The non hematological toxicities, as reported earlier,^16^ in the majority were mild, frequently reverted on continuing ATO and in the rest were reversible on discontinuing the drug for an interval of 1 – 2 weeks.^16^ As reported previously 5 (6.9%) cases developed a differentiation syndrome which resolved in 4 and was fatal in one. There were no sudden deaths attributable to a cardiac event in this series of patients and on long term follow up there were no cases with clinical cardiac dysfunction. There were no documented second malignancies documented in this cohort.^15^ Post remission induction, this regimen, was administered on an out patient basis. With the exception of some early reports of increased hepatic and cardiac toxicity^32^–^35^ the majority of subsequent reports using ATO in newly diagnosed cases is similar to our experience.^18^,^19^,^29^–^31^

There have been periodical major concerns raised about the administration of ATO. Very early there was a concern about cardiac arrhythmia related sudden deaths in patients with APL who were treated with ATO. Almost all these deaths happened in induction in previously heavily treated patients.^32^–^34^ There have been no such deaths reported when ATO was used for treating a number of other malignancies, albeit stable patients. Similarly, it does not appear when administered to patients with APL who are in remission (none reported in the literature). The role of QTc interval prolongation and limitations of the corrected QTc interval value generated with tachycardia due to any cause such as infection have been reviewed previously and it increasingly recognized and accepted that QTc prolongation is an electrocardiographic phenomenon with little clinical significance in the majority of patients.^36^ This does not mean we should not monitor it or ignore it, though response should be judicious and clinically appropriate. It has been reported that in more than 2900 cases treated by US-FDA approved ATO there have been no arrhythmia related deaths.^36^ We do not believe that there is anything sacrosanct or superior about the US-FDA approved ATO but rather that it in reality it was never a significant clinical problem beyond a few case reports where the arrythmia’s were probably related to other etiologies or at least in part contributed by them.

Next was the suggestion of acute hepatic failure and death from hepatoxocity occurred with ATO.^37^ There have been no other major reports since this initial publication about 12 years ago. This has definitely not been our experience with more than 250 patients treated to date at our center (newly diagnosed and relapsed).

There has always been a concern of second malignancies with the use of ATO. This is based on in-vitro experiments suggesting oxidative DNA damage^38^ and clinical observations from cases with long term environmental exposure. This theoretical concern is in contrast to clinical data available. In early reports of investigators from China it was noted that there was no increase in second malignancies in patients followed up for 10 years.^11^ A similar observation was made in 1982, in a cohort of 479 patients who had been treated with Fowlers solution [potassium arsenite] for duration varying from 2 weeks to 12 years during the period 1945 – 1969. The median cumulative dose in this cohort was 448 mg. It was noted that in this cohort of patients there was a marginal increase in fatal and non-fatal skin cancers but no increase in the incidence of internal malignancies.^39^

A recent report that was meant to highlight the low probability of ATO having induced cancers in patients (3 cases) receiving oral ATO^40^ was interpreted in a more recent review article as ‘highlights the concern of second malignancies’ in patients treated with ATO.^41^ To the best of our knowledge there are no other (if one insists on considering the previous report as second cancer to ATO therapy) reported cases of second cancers after administration of ATO at currently defined therapeutic doses. It would be reasonable at this point, though with limited long term follow up data, to state that second cancers following ATO are less than that reported following therapy of acute myeloid leukemia with currently accepted standard of care regimens.

There have been concerns raised about embryo toxicity based on animal models and some data from cases with environmental exposure.^42^ This again is not based on data in humans exposed to currently accepted therapeutic doses of ATO, this data for obvious ethical reasons is unlikely to be ever generated. However in our series, seven of the patients (4 women and 3 men) have had 8 normal babies,^15^ though all happened after completion of therapy. In this relatively young cohort there were no reports of abortions, fetal abnormalities or still births in any couple. While we did not actively evaluate fertility there were no reports of couples requesting evaluation for sterility.^15^

Hair and nail samples from 5 patients of this cohort who had completed therapy at least two years earlier was compared with that of 5 patients who had just completed therapy (not from this cohort) and 5 healthy controls. There was no significant difference in the ATO retention in hair and nail samples of controls and patients who had completed therapy at least two years earlier.^15^ The median levels, even among the patients who had just completed therapy was below the lower limit of the normal range described for normal controls by the Agency for Toxic Substances and Disease Registry (ATSDR based in Atlanta, Georgia, USA (http://www.atsdr.cdc.gov/).^15^

## Pattern and Timing of Haematopoietic Recovery Following Treatment with Single Agent Arsenic Trioxide

**Figure 3:**
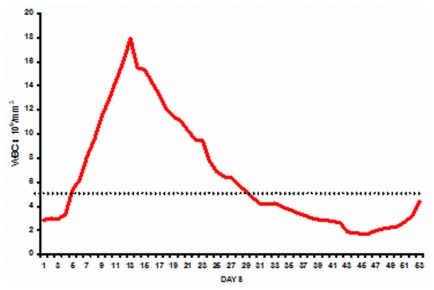
Average WBC count among patients with a leucocytic response and who achieved complete remission (n=6), illustrating the triphasic response.

**Figure 4:**
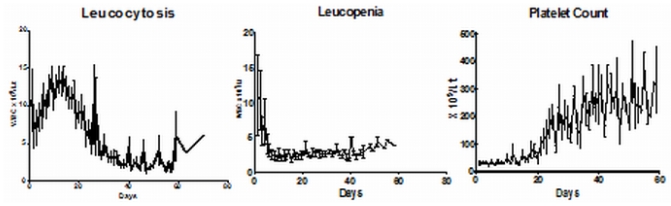
The mean WBC and Platelet count ± 1SE over time among patients treated on single agent ATO regimen. A)WBC response among those with leucocytosis (n=40). B) WBC response among those without leucocytosis (n=18). C) Platelet count recovery (n=60).

In our initial series the median time to achieve CR was 42 days (range: 24–70).^16^ However, this figure does not reflect the entire details of the kinetics of leukemia clearance and pattern of normal hematopoiesis recovery. As reported initially by us about two thirds of patients have a leucocytic response after initiation of ATO while in about a third there is prolonged leucopenia prior to gradual normalization.^43^ The leucocytosis can at times be very rapid and alarming and based on our early observations we had introduced hydroxyurea to control this leucocytosis with a recommended sliding scale to adjust the dose depending on the WBC count.^16^ We also noted that this was at times not adequate and we allowed use of an anthracycline in induction if there was rapid rise in the WBC counts after initiation of therapy at pre-defined levels and time points.^16^ In cases that there is leucocytic reponse there is often a second leucopenic phase (variable duration) and then recovery to normal values^43^ (a triphasic response; Figure 3). Unlike with the use of ATRA plus chemotherapy schedules the WBC count remains high (in two thirds) or very low (in one third), with a low platelet count and significant circulating promyelocytes for the first two weeks as illustrated in Figure 4. At this time point (around 2 weeks into therapy) there is often a concern raised, among those not familiar with this agent, as to whether ATO is doing anything at all to the disease and a consideration to change protocol or add on additional drugs is discussed. However, if the diagnosis is correct, with adequate support during this period and continuing ATO, all patients will go on to achieve CR. Another common observation in some cases is a clinically stable patient in the 4^th^ week of therapy, with a normal platelet count but very low WBC count and a consideration to stop ATO is made based on the argument that the ATO is causing a myelosuppresive effect. Our experience would suggest that ATO need not be stopped here but can be safely continued for the intended duration and one would probably be compromising treatment by prematurely stopping therapy at this time point.

## Pattern of Molecular Response Following Therapy with Arsenic Trioxide

**Figure 5:**
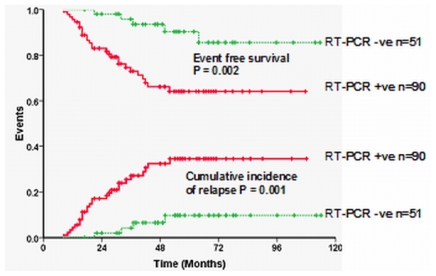
Event free survival and cumulative incidence of relapse based on RT-PCR positivity at the end of induction.

It is well recognized that the kinetics of leukemia clearance with the use of ATO, in induction, is significantly different from that of ATRA alone or ATRA plus chemotherapy combinations.^44^ It is not well studied or reported as to what the effects of ATO in consolidation and maintenance are on the kinetics of relapse. Extrapolation of data generated from ATRA plus chemotherapy regimens may potentially not be valid when applied to regimens that use ATO in upfront therapy. With conventional ATRA plus chemotherapy regimens it is well recognized that a positive RT-PCR at the end of induction did not have a significant prognostic effect while in those who were positive post consolidation had an increased risk of relapse.^41^,^45^ From our own data (unpublished, accepted for presentation at ASH 2011) a positive RT-PCR at the end of induction was the most significant risk factor for subsequent risk of relapse on a multivariate analysis (RR=4.9; 95% CI=1.13–21.20; P-value=0.034) and a positive RT-PCR at this time point had a sensitivity of 86.7% and specificity of 42.3% for predicting relapse (Figure 5). In this single agent low intensity ATO regimen the presence of a positive RT-PCR at the end of induction could potentially help identify a subset of patients who may benefit from intensification of this regimen. This strategy remains to be validated in a large clinical trial.

## Impact of Additional Cytogenetic and Molecular Markers Such as FLT3 Mutations on Clinical Response Following Treatment with Arsenic Trioxide

The presence of cytogenetic abnormalities at diagnosis remains an important prognostic variable in patients with newly diagnosed AML.^46^ Secondary cytogenetic changes have been reported to have an adverse impact in some subsets of AML, though in patients with APL treated with conventional chemotherapy a similar adverse effect was not reported.^47^,^48^ We initially reported a small series of newly diagnosed patients with APL treated with single agent ATO in which our analysis suggested that there was no significant adverse impact of the presence of an additional karyotypic abnormality at diagnosis^49^. However, a more recent analysis of our data (larger cohort) does suggest that there may be an adverse impact of an additional cytogenetic finding at diagnosis in newly diagnosed patients though it was not significant on a multivariate analysis (unpublished data).

Fms-like tyrosine kinase 3 (FLT3) is a member of the class III receptor tyrosine kinase family and is expressed on haematopoietic progenitors.^50^,^51^ Mutations in the FLT3 receptors have been reported to be associated with a poor prognosis in both adult and paediatric patients with a diagnosis of acute myeloid leukaemia (AML).^50^ Mutations in the FLT3 receptor are commonly seen in patients with APL^50^. The common activating mutations of FLT3 in leukaemia include the FLT3 internal tandem duplication (FLT3-ITD) and a point mutation in the activation loop (D835V).^50^ A recent gene expression profiling study reported that patients with APL could be segregated into those with and without a FLT3-ITD mutation, suggesting that these groups were biologically different.^52^ A retrospective analysis of the impact of FLT3 mutations in patients with APL, treated with conventional ATRA plus chemotherapy regimens, reported a higher incidence of induction deaths in one study^53^ while another study reported a trend towards a shorter overall survival.^54^ More recently Chillon et al^55^ analyzed the Spanish co-operative group data and showed that ppatient’s with increased ITD mutant/wild-type ratio or longer ITD size displayed shorter 5-year relapse-free survival (RFS) (P=0.048 and P<0.0001, respectively), though patients with D835 mutations did not show differences in RFS or overall survival (OS). In our series we found that FLT3-ITD mutation in 21% and its presence did not impact the clinical outcome of patients treated with ATO.^49^ We did however note a longer time to molecular remission among those who were FLT3 mutation positive.^49^

## Arsenic Trioxide for the Treatment of Relapsed Acute Promyelocytic Leukemia

Patients who relapse following an ATRA based chemotherapy schedule can achieve a second CR in 60 – 95% of cases with chemotherapy though the toxicity with such a regimen in this population approaches that seen with high dose chemotherapy for acute myeloid leukemia.^36^ There is a high incidence of ATRA resistance in this population especially if the relapse occurred within a year of completing an ATRA based chemotherapy schedule. In this setting ATO is extremely effective in inducing molecular remissions in the majority of patients without the toxicity profile of combination chemotherapy and does not have cross resistance with ATRA.^36^ This is the only indication for which it is approved by the United States Food & Drug Administration (FDA). Achieving molecular remission prior to a consolidation with an autologous stem cell transplant, the preferred option in this setting, has a significant bearing on long term outcome. Use of single agent ATO as consolidation therapy after achieving molecular remission was less effective in this population with a 2 year OS of 41% in one series^36^ and an EFS of 33% in a second.^37^ In our own series we reported a significantly better clinical outcome in patients who were consolidated with an autologous SCT versus those consolidated with ATO or ATO+ATRA following treatment of a frank hematological relapse of APL.^56^ Based on the available data it would be reasonable in patients with a hematological relapse to induce molecular remission with ATO and consolidate with an autologous SCT (SCT) in those who achieve molecular remission and consider an allogeneic SCT for those who fail to achieve a molecular remission.

## Conclusions

While there is no doubt as to the efficacy of ATO in the management of APL, its position in the algorithm in the treatment of this condition is still being defined for newly diagnosed cases. Based on the available data, it is clear that as a single agent it is the most effective drug in the management of APL. For patients who have relapsed following conventional ATRA plus chemotherapy regimens it is the established agent of choice to induce a second molecular remission. Preliminary concerns of fatal toxicity profile appear to be related more to the associated co-morbid conditions than the drug itself, as noted by their absence when used in patients with newly diagnosed APL without co-morbid conditions and in other malignant conditions. On going concerns that are frequently voiced about potential long term toxicity are not based on significant data. Better understanding of its in-vivo biotransformation and effect of the different methylated derivatives that are generated in this process, could potentially help further reduce its toxicity profile while enhancing its efficacy. This could be achieved by better ability to predict toxicity or efficacy based on genetic polymorphisms that have an impact on biotransformation pathways or by the use of specific methylated derivatives for therapy rather than the native compound which further research may potentially demonstrate to have a more favorable therapeutic profile.

In the developing world where the cost of ATO (generic) is low, the absence of grade III/IV neutropenia and mucositis along with the ability to administer the regimen on an out patients basis post remission induction significantly reduces the cost of treating this condition in comparison to a standard ATRA plus chemotherapy regimen. In summary there is sufficient evidence to embrace ATO as an important drug in the armamentarium for the treatment of APL, the optimal mode, combination and schedule for utilization of this agent in newly diagnosed cases remains to be defined.

## Figures and Tables

**Table 1:** Mechanism of action of arsenic trioxide.

I] Induce apoptosis (0.5–1.0μM)	II] Induce differentiation ( <0.5μM)
-downregulation of bcl2^5^	-degradation of PML-RARα^57^
-increased expression of caspases^58^	- methylation of histones^59^
-activation of jun kinases^60^	
-reorganize PML oncogenic domain (POD)^61^,^62^	
-disruption of cytoskeleton^63^	
-inhibition of NFkB^64^,^65^	
**III] Altered cellular Redox status**	**IV] Inhibits angiogenesis**
-reactive oxygen species (ROS) generation^67^,^68^	-down regulates VEGF^66^
-bind sulfhydryl rich proteins/enzymes such as glutathione and reduce there levels^69^	

**Table 2:** Summary of studies using arsenic trioxide in front line therapy in the treatment of APL.

	N	CR	EFS/DFS
**Mathews et al.**^15^	72	86%	5 year EFS 69% 5 year OS 74%

**Ghavamzadeh et al.**^18^	197	86%	5 year OS 64% 5 year DFS 67%

**Hu et al.**^31^ (+ATRA*+chemo)	85	94%	5 year EFS 89% 5 year OS 92%

**Ravandi et al.**^29^ **(+ATRA, +GO**#**)**	82	91%	3 year OS 85%

**Niu et al.**^37^	11	73%	1 year OS 73%

**Powell et al.**^70^	244	NA@	3 year EFS 80%
	(RCT$ ATO post induction)		3 year OS 86%

**Gore et al.**^30^	45	NA	3 year EFS 76%
	(ATO post induction)		3 year OS 88%

* ATRA – all-trans retinoic acid.

# GO – gemtuzumab.

@ NA –not applicable.

$ RCT – randomized controlled trial.
